# Deep Learning Classification of Treatment Response in Diabetic Painful Neuropathy: A Combined Machine Learning and Magnetic Resonance Neuroimaging Methodological Study

**DOI:** 10.1007/s12021-022-09603-5

**Published:** 2022-08-26

**Authors:** Kevin Teh, Paul Armitage, Solomon Tesfaye, Dinesh Selvarajah

**Affiliations:** 1 Academic Unit of Radiology, Department of Infection, Immunity and Cardiovascular Disease, University of Sheffield, Sheffield, UK; 2 Diabetes Research Department, Sheffield Teaching Hospitals NHS Foundation Trust, Sheffield, UK; 3 Department of Oncology and Metabolism, University of Sheffield, Sheffield, UK

**Keywords:** Functional magnetic resonance imaging, Resting state, Convolutional neural network, Painful diabetic peripheral neuropathy, Treatment response

## Abstract

Functional magnetic resonance imaging (fMRI) has been shown successfully to assess and stratify patients with painful diabetic peripheral neuropathy (pDPN). This supports the idea of using neuroimaging as a mechanism-based technique to individualise therapy for patients with painful DPN. The aim of this study was to use deep learning to predict treatment response in patients with pDPN using resting state functional imaging (rs-fMRI). We divided 43 painful pDPN patients into responders and non-responders to lidocaine treatment (responders n = 29 and non-responders n = 14). We used rs-fMRI to extract functional connectivity features, using group independent component analysis (gICA), and performed automated treatment response deep learning classification with three-dimensional convolutional neural networks (3D-CNN). Using gICA we achieved an area under the receiver operating characteristic curve (AUC) of 96.60% and F1-Score of 95% in a ten-fold cross validation (CV) experiment using our described 3D-CNN algorithm. To our knowledge, this is the first study utilising deep learning methods to classify treatment response in pDPN.

## Introduction

The application of machine learning (ML) methods to the analyses of neuroimaging datasets has led to significant advances in the field. It has enabled a shift from population/cohort-based analyses into more individualised biomarkers of disease or functional brain states. From a clinical perspective, this has fundamental importance in diagnosis, prognosis and patient stratification. One ML approach that is increasingly being used on imaging datasets to detect lesions (Zhang et al., 2019b), automate tissue segmentation (Liu et al., 2018) and classify different brain disorders (Farooq et al., 2017) is deep learning using convolutional neural network (CNN). One thing in common across these different applications is the identification of prominent delineated features on imaging datasets and there have been numerous studies investigating the utility of CNN in this context. However, the classification of clinical phenotypes using raw medical images in the absence of well-defined delineated features poses substantial challenges for CNN and has not been fully investigated.

In this study, we will examine a well-characterised cohort of patients with chronic neuropathic pain secondary to diabetes. Neuropathy is one of the commonest complications of diabetes causing disabling pain in the lower and upper limbs in up to a quarter of patients. This often results in considerable disability and suffering. Pharmacotherapy is the mainstay of treatment to alleviate symptoms but it is often ineffective. Even with optimal pharmacotherapy, only a third of patients experience any meaningful pain relief (i.e. a 50% reduction in pain intensity scores) (Finnerup et al., 2015; Katz et al., 2008). We recently demonstrated that combined ML and magnetic resonance (MR) imaging could accurately predict patients into sensory phenotypes (Teh et al., 2021) with an accuracy of 0.92. This has the potential to serve as a biomarker for use in patient stratification, leading to a more efficient treatment approach. We used a linear, support vector machine (SVM) algorithm using features extracted from rs-fMRI and MRI volumetry.

Independent component analysis (ICA) is an effective method to derive brain functional networks with both functional connectivity and spatial information (Nickerson et al., 2017; Rajapakse et al., 2006). Unlike commonly used seed based analysis approaches (seed to voxel and ROI to ROI), ICA is a pure data-driven method, which generates highly reproducible large-scale brain networks (Damoiseaux et al., 2006). A previous study using gICA analysis has shown that resting state functional connectivity can successfully predict subsequent duloxetine treatment response of major depressive disorder (Fu et al., 2015). To capture these elusive feature delineations, 3D group ICA features have been shown to achieve superior classification accuracy (Qureshi et al., 2019) when used in conjunction with CNN techniques.

Hence, the primary aim of this ML study is to train a CNN-based classification model to differentiate responders and non-responders to neuropathic pain treatment using resting state (RS) maps as inputs to a 3D-CNN architecture. The secondary aim was to compare the classification performance of two different inputs (all group-level ICA components vs statistically significant group-level components) for the 3D-CNN architecture.

## Method

### Study Design and Participants

Forty-three consecutive patients (responders n = 29 and non-responders n = 14) with painful diabetic neuropathy were recruited for this study. We used intravenous (IV) lidocaine as a experimental model to examine treatment response in this neuroimaging study. It is a recognised treatment option in specialist centres for the management of intractable neuropathic pain i.e. when conventional treatment is ineffective (Kastrup et al., 1986, 1987; Viola et al., 2006) and included in clinical guidelines (Tesfaye et al., 2011). It is also a good experimental model for assessing neuroimaging markers of treatment response because 1) it has a central mechanisms of action (Abdi et al., 1998; Devor et al., 1992; Omana-Zapata et al., 1997); 2) a short half-life (40 min) – nullifying a direct treatment effect on MRI measures; and 3) up to one half of patients do not respond to treatment—enabling assessment of matched groups of responders and non-responders. Patients completed a standardised questionnaire to determine treatment response to IV lidocaine treatment. Responders were defined as patients who report at least a 30% reduction in pain intensity score (0 to 10 numeric rating scale, where 0 = no pain and 10 = worse pain imaginable) post lidocaine treatment. Study visits were conducted prior to IV lidocaine treatment. Altogether, there were two study visits: Visit 1 was for detailed clinical and neurophysiological assessments and Visit 2 for MR neuroimaging.

Details of the clinical and neurophysiological assessment performed at Visit 1 are shown in Table 1. There were no statistically significant differences between responders and non-responders in the assessments performed. Written informed consent for the study was obtained before subjects participated in the study, which has prior approval by the Sheffield Local Research Ethics Committee (Sheffield, U.K.).

**Table 1:** Demographic, metabolic and neurophysiological assessments of study participants

	**Responder**	**Non Responder**	**p**
**n**	29	14	
**Age (years)**	57.48(8.82)	57.07(11.12)	0.896
**Male sex (n, %)**	19, 66	5,36	
**Type of diabetes (Type 1/2)**	9,20	3,11	
**Duration of diabetes (years)**	20.41(14.7)	20.86(14.6)	0.926
**Duration of pain (years)**	10.37(7.51)	7.89(6.34)	0.292
**Hba1c (mmol/mol)**	70(18.42)	73(16.67)	0.714
**NTSS-6 score**	16.01(3.29)	14.18(4.29)	0.176
**TCNS**	21.19(4.49)	16(9.26)	0.07
**BMI**	33.23(11.46)	30(5.6)	0.328
**Becks**	23.84(13.06)	11.71(14.15)	0.051
**DN4**	7.86(1.49)	6.67(1.58)	0.055
**Sural**			
Conduction velocity (m/s)	19.12(17.03)	14.98(18.73)	0.562
Amplitude (mAmp)	3.98(6.18)	1.9(5.7)	0.392
**Common Peroneal Nerve**			
Conduction velocity (m/s)	26.35(17.97)	29.75(20.22)	0.646
Amplitude (mAmp)	2.04(2.2)	1.39(2.29)	0.467
Distal latency (ms)	3.73(2.83)	4.2(3.27)	0.693
**Tibial Latency**	3.7(2.94)	4.07(3.46)	0.829

Data presented as mean (standard deviation) unless otherwise stated. Groups were compared using Student’s t-tests

*NTSS-6* Neuropathy Total Symptom Score-6 pain questionnaire, *TCNS* Toronto Clinical Neuropathy Score, *DN4* Douleur Neuropathique en 4 Questions

### MRI Acquisition

On the second visit prior to IV lidocaine treatment subjects underwent MRI using a Phillips Achieva 3 Tesla system (Phillips Medical Systems, Holland) with a 32-channel head coil. T1-weighted magnetisation prepared rapid acquisition gradient echo sequence was used to acquire anatomical data with the following parameters: repetition time (TR) 7.2 ms, echo time (TE) 3.2 ms, flip angle 8°, and voxel size 0.9 mm3, yielding isotropic spatial resolution. Resting-state fMRI data was also acquired while subjects fixated on a cross using a T2*-weighted pulse sequence, with TE = 35 ms; TR = 2600 ms, in-plane pixel dimensions = 1.8 mm × 1.8 mm, contiguous trans-axial slices thickness of 4 mm, a slice acquisition order = ascending (bottom-up), FOV = 128 × 128 mm, pixel bandwidth of 2129, number of phase encoding steps of 94 and an echo train length of 53.

### Pre-processing and Data Analysis

Group ICA based analysis was performed using the CONN (version 18.b) (Whitfield-Gabrieli & Nieto-Castanon, 2012): functional connectivity toolbox software which was also used to perform all pre-processing steps (using the default pre-processing pipeline), as well as subsequent statistical analyses, on all subject scans. Using the CONN toolbox pre-processing pipeline, raw functional images were slice-time corrected, realigned (motion corrected), unwarped, and co-registered to each subject's T1-weighted dataset in accordance with standard algorithms. Images were then normalised to Montreal Neurological Institute (MNI) coordinate space, spatially smoothed (5 mm full-width at half maximum), and resliced to yield 2 × 2 × 2 mm voxels and the resting state connectivity analysis was performed using CONN toolbox. Further resting state analysis using gICA in CONN toolbox was performed with variance normalisation pre-conditioning, subject or condition concatenation of BOLD signal data along temporal dimension, group level dimensionality reduction, fast ICA for estimation of independent spatial components, and GICA1 back-projection for individual subject-level spatial map estimation (Beckmann et al., 2009). The number of independent components to be estimated was set to 30 and dimensionality reduction was set to 64.

### Proposed Framework

**Fig. 1:**
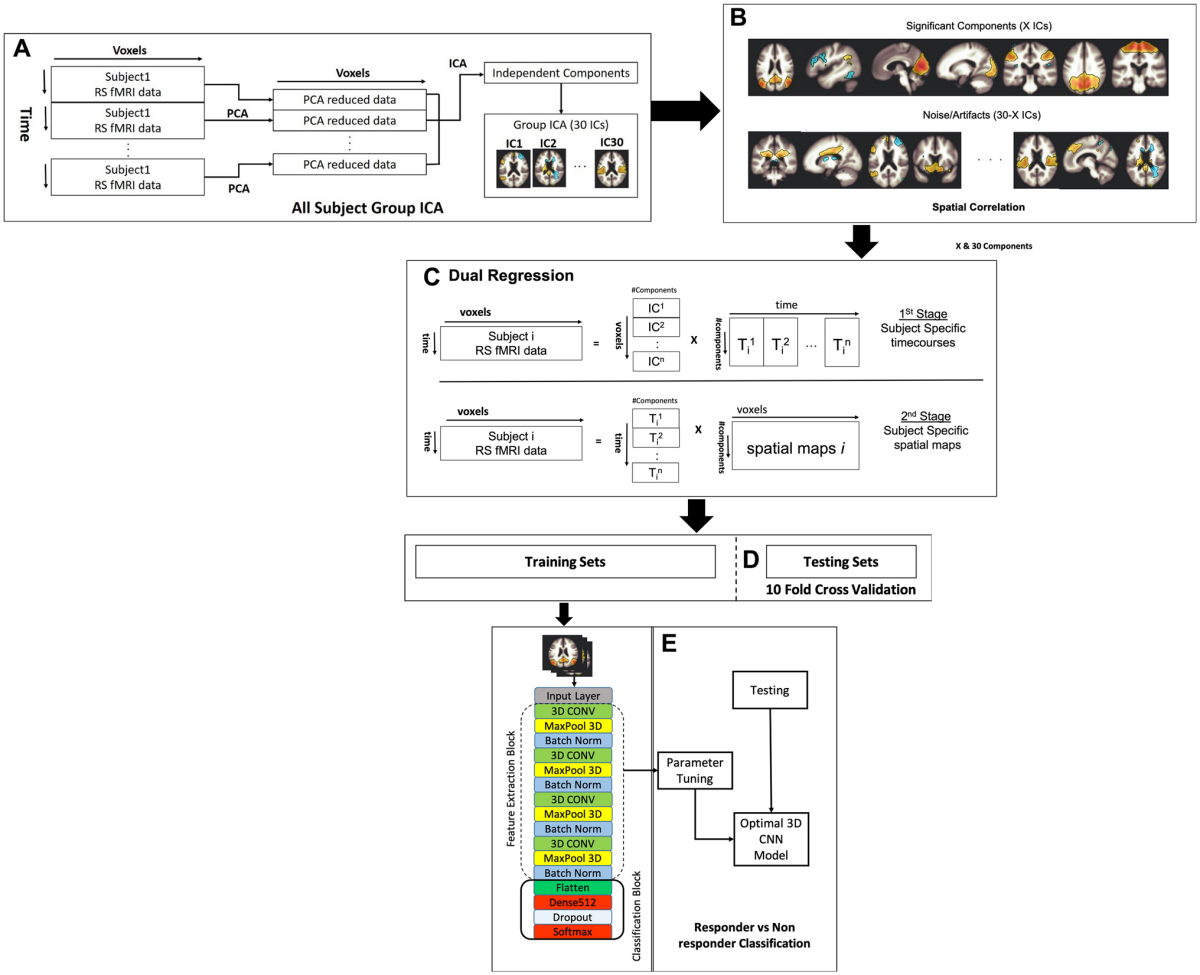
Illustration of our proposed treatment response classification framework using group ICA features extracted from rs-fMRI scans. Firstly, (**A**) shows the 30 group-level ICA networks from all studied subjects. Next, we used CONN toolbox spatial to match template-clustering tool to identify the useful functional group-level ICA maps. As a result, 8 IC selected spatial components (SSC) (p < 0.1) were identified as statistically useful networks whereas 22 ICs were not used in the selected IC subset (**B**). Then these group-level components (8 and 30 IC components) were used to extract subject-specific time courses (stage 1) and subject-specific spatial ICA maps (stage 2) using dual regression method in (**C**). Step (**D**) presents the nested tenfold cross validation strategy to train the classifier and regressors, tune the hyperparameters and test their performances. Next the 4D ICA spatial component maps (concatenated multiple 3D spatial component maps) were fed into the 3D-CNN (**E**) for treatment response classification

Figure 1 illustrates the procedures used for joint prediction of treatment response status in this study. Figure 1a shows the first step of the procedure in which the group-level 3D ICA spatial maps of all studied subjects were computed. Next, we employed an automatic clustering tool (using CONN toolbox spatial match to template) to identify the useful group-level independent components (X ICs) for further analysis; we also compared all analysed components (30 ICs). We then performed the two-stage dual regression (Fig. 1c) to extract: 1) subject-specific IC time courses (stage 1) and; 2) subject-specific ICA spatial maps (stage 2). Figure 1d presents the nested tenfold cross validation (CV) strategy used to evaluate the performance of the proposed framework. The subject-specific ICA maps were applied to a 3D Convolutional Neural Network (3D-CNN) for the classification task (Fig. 1e).

### Deep Learning Features

Multiple 3D spatial component maps were acquired by performing dual regression (Beckmann et al., 2009) on the group ICA. The 3D spatial maps of these selected functional networks were then inputted into our chosen 3D-CNN for classification of responders and non-responders to IV lidocaine treatment groups. This work examine differences in classification performance between an approach using all ICA spatial components (ASC) and another using a semi-automatically selected meaningful independent component time-series dataset (SSC). There were 8 spatial maps (SSC) identified when matched to a spatial template in CONN toolbox chosen with p < 0.1. These 8 chosen maps will be shown in more detail in the results section. We also compared the classification results of the 8 selected spatial component maps with all 30 spatial component maps. Non post-processed resting state pre-processed image inputs were also used as a base comparison.

### Deep Learning and 3D-CNN Framework

We used a 3D-CNN based deep learning classification framework in this study based on a lightweight Voxnet (Maturana & Scherer, 2015) architecture. The framework was implemented on the TensorFlow library version 2.5 with two bridged Nvidia Ge-Force GTX 2080Ti graphical processing units (GPU). For the training model, we used the Adam optimizer with a learning rate of 0.001 and setting epsilon at 0.1. Since the size of the dataset was relatively small for deep learning, to avoid model overfitting, we used ten-fold cross-validation in this study to report the mean performance of the overall model.

A modified version of the Voxnet classification framework was used in this study. Specifically, we added batch normalization layers in the convolution layer. A dropout rate of 0.3 was used in the fully connected layers with a batch size set at 4 and using 100 epochs. The parameters including learning rate, epsilon value, dropout rate, batch size, and epoch size were optimised using the following ranges. For epsilon, we tuned it in the range of [0.1: 0.05: 1], for learning rate, we tuned it in the logarithmic range of [1, 0.1, 0.01, 0.001, 0.0001, and 0.00001], for the dropout rate, we tuned it in the range [0.1: 0.05: 1], for the batch size, we optimized it by the maximum available GPU memory, and the number of epochs were tuned in the range of [10: 1: 200]. We used a binary entropy loss function when training the deep learning network, and an early stopping criterion was used to stop the network training with respect to the leave-one-out cross-validation loss. The optimal parameter tuning was chosen to reflect the best performance metrics for the ASC and SSC dataset. The chosen parameters were similarly used on the pre-processed resting state image data (RSP).

### Performance Measures

One of the most crucial processes of defining a machine learning model is model evaluation. For this work, we will compare standard metrics such as accuracy (ACC), precision (PR) and recall (RE) with average cross-validation AUC and F1-Score. We choose to include the AUC and F1-score due to the slight imbalance in our dataset leaning towards treatment responders to lidocaine. Of all these performance metrics we regard the AUC and F1 score to be the most important metric in this work. Previous work has concluded that these measures fit best for imbalanced data problems (Jeni et al., 2013; Raeder et al., 2012). These, two performance measures are described further. Firstly, introducing some acronyms TP, TN, FP and FN are the number of true positive (responders), true negative (non-responders), false positive and false negative samples, respectively. The metrics used are shown below:

1 $$ACC= \frac{TP+TN}{TP+TN+FP+FN}$$

2 $$PR= \frac{TP}{TP+FP}$$

3 $$RE=\frac{TP}{TP+FN}$$

where precision measures samples correctly classified as positive, and recall describes the proportion of all positive samples classified as positive. F1 score is interpreted as a weighted average of the precision (PR) and recall (RE):

4 $$F1=2\cdot \frac{PR.RE}{PR+RE}$$

AUC score (Area Under the receiver operating characteristic Curve) characterises the area under the curve of sensitivities plotted against corresponding false positive rates (FPR):

5 $$FPR=\frac{FP}{FP+TN}$$

## Results

### Classification Results

Our results suggest that using ICA analysis specifically spatial component maps can be used as a good discriminatory predictor of lidocaine treatment response in painful DPN patients. In our study, we compared using ASC, SSC and RSP as the input to our CNN classification workflow. Comparing the classification performances, the first thing we noticed from Table 2 is the sub optimal results obtained from the pre-processed resting state data. We achieved a F1-Score of 69% and an AUC score of 44%. On the other hand, ASC gave the best performing results achieving a F1-Score of 95% and a mean balanced AUC of 96.60% in a ten-fold CV experiment using the described 3D-CNN algorithm. As compared to SSC it also achieved good performance with F1-Score of 90% and AUC of 85%. The rest of the results for the three groups are shown in Table 2.

**Table 2:** Described performance metrics comparing ASC and SSC results

	**ASC**	**SSC**	**RSP**
**Accuracy**	0.9307	0.8529	0.6743
**Precision**	0.9237	0.8460	0.6744
**Recall**	0.9787	0.9623	0.7136
**F1-Score**	0.9504	0.9004	0.6934
**AUC**	0.9661	0.8499	0.4375

Also, when running our networks, we also calculated the timing cost comparison. Using the deep learning configuration described above with our lightweight modified Voxnet CNN pipeline took 3.4 min to train 100 epoch per fold for the SSC, 5.1 min for ASC and 29.3 min for RSP.

### Network Component Analysis

Table 3 shows the ranking of each functional network as the features of a deep learning framework. These components were chosen based on p < 0.1. The uncorrected *p*-value revealed the component’s significance. Using this significance level we identified eight ICA spatial components from a subset of 30. The 5 resting state functional networks identified by the CONN network cortical ROIs (HCP-ICA) through the ICA analysis were the cerebellar network (CEB), default mode network (DMN), frontoparietal (FPN), sensorimotor network (SMN) and visual network (VN). They were used to perform dual regression to generate subject-specific time courses for connectivity analysis and spatial maps for classification.

**Table 3:** SSC networks selected based on comparison of network activities between responders to lidocaine and non-responders at p < 0.1

**Component Name**	**Component Number**	**p-value**	**Pearson(r)**
Cerebellar	ICA1	0.014	0.373
Default Mode	ICA10	0.003	0.446
FrontoParietal	ICA5	0.058	0.292
Sensorimotor	ICA9	0.099	0.254
Sensorimotor	ICA26	0.058	0.291
Sensorimotor	ICA29	0.026	0.339
Visual	ICA6	0.009	0.394
Visual	ICA11	0.001	0.579

## Discussion

To the best of our knowledge, this is the first study classifying treatment response using rs-fMRI and 3D-CNN deep learning architecture. The key findings of this work are 1) using ICA spatial component maps (ASC and SSC) performs better than only using RSP as the input to our CNN networks; 2) using all the group ICA spatial components (ASC) information performs better compared to the semi-automatic selection of the highly relevant networks (SSC) and 3) a lightweight 3D-CNN deep learning architecture for classification uses imaging data more efficiently. With these approaches, we achieved better treatment response classification results.

**Fig. 2:**
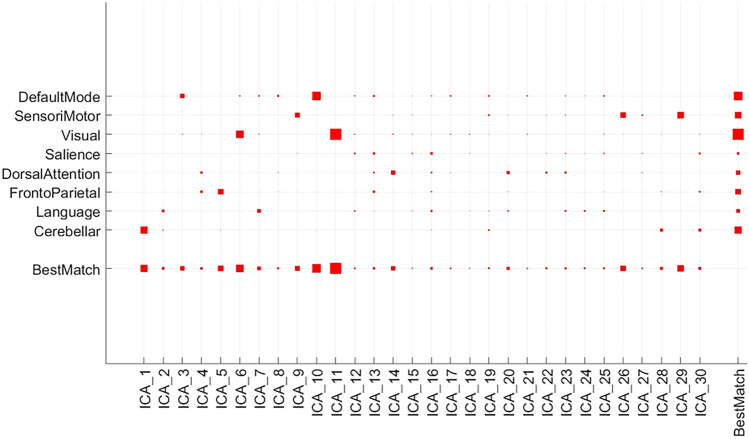
Spatial to match template (CONN toolbox gICA) to select the best SSC based on p < 0.1

Our analyses show a clear disparity between RSP and both the ICA analysed spatial component maps (ASC and SSC) giving an enhanced performance score. When comparing ASC and SSC there is an improvement for the ASC score with a 5% improvement in the F1-Score and a 11.6% improvement for the AUC. The extra 22 spatial components in the ASC which are noisier (not satisfying the p < 0.1 condition of the 8 networks shown in Fig. 2) is shown to have contributed to the performance metric boost of the ASC method. Using deep learning we were able to automatically discover the intricate structure differences of the responders and non responders imaging features especially true when larger imaging information was used during the training step as shown by (LeCun et al., 2015). Hence from our results we surmise that when a dataset is closely matched (in our case painful diabetic neuropathy patients) using more ICA components provides better performance. In future we will explore SSC classification performance more using different p value network selection (i.e. p < 0.2, p < 0.3 etc.) to further our assertion.

We did not explicitly compare our Voxnet based lightweight model with other popular deeper learning networks such as Resnet or VGG-16 (Simonyan & Zisserman, 2014). We calculated the trainable parameters of VGG-16 to be around 2.5 billion compared to around 1.4 million using Voxnet with identical 4D ICA spatial component input dataset. Hence due to GPU memory limitations, we did not compare our proposed CNN model with these deeper networks. When the computation time was compared between SSC and ASC there was an extra 1.7 min computation time per fold when using the ASC as the input data. We consider this a good use of extra computational time given the boost of performance when using the ASC input dataset.

In our literature search, we did not find any treatment response classification of pDPN using machine learning. We, however, found two closest studies focusing on pain and treatment response classification using ML. The first study used a SVM classifier to differentiate lower back pain and healthy volunteers (Lamichhane et al., 2021). They achieved an average classification accuracy of 74.51% and an AUC = 0.787. The second study used deep learning (DL) neural networks in classifying chronic pain patients and pain-free controls (Santana et al., 2019). Their best model using the Ann4brain architecture and MSDL parcellation had a balanced accuracy of 86.8% and AUC of 0.918. Due to the differences in patient cohorts, we cannot directly compare these results with ours. However, with our high testing accuracy of 93% and AUC of 97%, we believe we can translate our painful DPN CNN model to other disease cohorts. For example, with other neuropathy patients (i.e. idiopathic and chemotherapy painful neuropathy).

Another important factor in our study is that we set the number of ICA components to 30, as is conventionally used in a recent study (Qureshi et al., 2019). Recently, it has been shown that the detected disease-related differences in functional connectivity may alter as a function of ICA model order, specifically how many ICA components to use (Abou Elseoud et al., 2011). Their multi-level ICA exploration of unmedicated seasonal affective disorders functional connectivity enables optimisation of sensitivity to brain disorders and found 45 RS networks as the optimum component number to use. To further explore the effect of chosen RS ICA components, a recent study compared 2, 10 and 45 ICA chosen components. They reached an accuracy of 95% for the 2 components, 93% for the 10 components and 92% for the 45 components. In our study, the performance decreases with smaller component number, which is the opposite of the described study. We will investigate a wider number of set ICA component analysis to further affirm our findings.

Our study also used gICA, a data driven method, to investigate the association between brain networks. Here, we decomposed the signal from whole brain voxels to spatially and temporally independent components. Future work will involve using different indices of functional brain alterations, including amplitude of low-frequency fluctuations (ALFF), regional homogeneity (ReHo), and regional functional correlation strength (RFCS) which has been successfully demonstrated in a CNN migraine study (Yang et al., 2018). In our gICA analysis method, we only compared the analysis of SSC and ASC. Here, we concatenated the 3D spatial components together (8 and 30), which consists of different resting state networks (RSN). However, in future to fully understand and interpret our results further we will also explore resting state networks individually between our two groups. Of particular interest to our pDPN dataset are the default mode, sensorimotor and fronto parietal networks. This will enable us to identify and rank individual networks based on the binary classification performance as shown in a recent work on Autism spectrum disorder (Yang et al., 2021). To further this study we will analyse the resting state data with higher regional sensitivity using a region of interest (ROI) to ROI analysis approach. The outcome of the ROI-ROI analysis will also be expanded further with the use of linear classifiers for example linear SVM instead of black box models like deep learning for better interpretability as shown recently (Teh et al., 2021).

This study has some limitations. Our dataset of 43 samples is relatively small. However, in our previous study, we have shown with similar sample sizes using resting state ROI to ROI image analysis to have enough sample size power to clearly differentiate between lidocaine treatment responses (Wilkinson et al., 2020). Another limitation is the lack of a separate testing dataset. To address this, the results presented are the ten-fold cross-validated performance metrics of our CNN method. In future work, we intend to apply our algorithm to other treatment response datasets to externally validate our algorithm. This will include out of sample testing with within disease datasets (painful DPN) and also between disease datasets (idiopathic and chemotherapy induced neuropathy). In addition, although we have shown that a 3D-CNN model trained on rs-fMRI datasets accurately classifies patients according to their treatment response (i.e. based on analgesic response), we are unable to determine or explore the impact of neuropathy or diabetes itself on cortical changes described. To address this, future studies should consider including appropriate control subjects (e.g. participants with diabetes and no neuropathy or subjects with neuropathic pain from different aetiologies).

In summary, in the last few years CNNs have been used very successfully with rs-fMRI to classify different disease phenotypes (Qureshi et al., 2019). We have demonstrated the effectiveness of using a 3D lightweight CNN method, which not only saves on computational time but also gives high prediction scores. We believe that the presented results show that rs-fMRI has great application potential as a biomarker in neuropathic pain (Cauda et al., 2009, 2010; Sağ et al., 2019; Zhang et al., 2019a). Finally, we have successfully shown that we are able to differentiate responders and non-responders to neuropathic pain treatment using 3D gICA resting state (RS) maps as inputs to a 3D-CNN architecture.

## Information Sharing Statement

The analysed RS data that supports the findings of this study can be downloaded at https://doi.org/10.5281/zenodo.6325587. Pre-processed or raw resting state data associated with this work can be provided upon request.
